# Sesame Seeds: A Nutrient-Rich Superfood

**DOI:** 10.3390/foods13081153

**Published:** 2024-04-10

**Authors:** Parisa Mostashari, Amin Mousavi Khaneghah

**Affiliations:** 1 Department of Food Science and Technology, Faculty of Nutrition Sciences and Food Technology, National Nutrition and Food Technology Research Institute, Shahid Beheshti University of Medical Sciences, Tehran 1981619573, Iran; mostashari.p.63@gmail.com; 2 Department of Food Science and Technology, Faculty of Pharmacy, Tehran Medical Sciences, Islamic Azad University, Tehran 1941933111, Iran; 3 Faculty of Biotechnologies (BioTech), ITMO University, 9 Lomonosova Street, Saint Petersburg 191002, Russia

**Keywords:** sesame (*Sesamum indicum* L.), sesame oil, bioactive compounds, health benefits, extraction methods

## Abstract

Sesame seeds (*Sesamum indicum* L.) have been cultivated for thousands of years and have long been celebrated for their culinary versatility. Beyond their delightful nutty flavor and crunchy texture, sesame seeds have also gained recognition for their remarkable health benefits. This article provides an in-depth exploration of the numerous ways in which sesame seeds contribute to overall well-being. Sesame seeds are a powerhouse of phytochemicals, including lignans derivatives, tocopherol isomers, phytosterols, and phytates, which have been associated with various health benefits, including the preservation of cardiovascular health and the prevention of cancer, neurodegenerative disorders, and brain dysfunction. These compounds have also been substantiated for their efficacy in cholesterol management. Their potential as a natural source of beneficial plant compounds is presented in detail. The article further explores the positive impact of sesame seeds on reducing the risk of chronic diseases thanks to their rich polyunsaturated fatty acids content. Nevertheless, it is crucial to remember the significance of maintaining a well-rounded diet to achieve the proper balance of n-3 and n-6 polyunsaturated fatty acids, a balance lacking in sesame seed oil. The significance of bioactive polypeptides derived from sesame seeds is also discussed, shedding light on their applications as nutritional supplements, nutraceuticals, and functional ingredients. Recognizing the pivotal role of processing methods on sesame seeds, this review discusses how these methods can influence bioactive compounds. While roasting the seeds enhances the antioxidant properties of the oil extract, certain processing techniques may reduce phenolic compounds.

## 1. Introduction

Sesame (*Sesamum indicum* L.) is a plant classified under the Pedaliaceae family and is often called the “seed of immortality.” This plant is an erect annual herb that has different names across various cultures, including *ajonjoli* (Spanish), *hu ma* (Chinese), *gergelim* (Portuguese), *goma* (Japanese), *sesame* (French), *til* (Hindi), and *konjed* (Persian) [1,2,3]. This crop is among the earliest to have been domesticated for oil production and also served as one of the first condiments utilized [4,5]. The precise location of sesame’s domestication remains uncertain; however, despite various assertions, it is widely believed that the crop originated in Africa and subsequently disseminated to West Asia, China, India, and Japan [5,6]. The harvested area (which has expanded from 5.0 million hectares in 1961 to 13.97 million ha in 2022) and the production of sesame have increased over the last few decades (1.4 million tonnes in 1961 to 7.4 million tonnes in 2022) [7,8]. Despite its widespread cultivation in various regions of the southern United States, Latin America, Asia, and Africa, the crop commonly known as the “queen of oilseeds” is considered an orphan crop and is not currently mandated by any International Crop Research Institute for Semi-Arid Tropics [7,9]. According to the Food and Agriculture Organization, most of the world’s sesame crop is cultivated in less developed nations like Uganda, Sudan, Nigeria, India, China, Burma, and Brazil. South Sudan ranks fifth in the world for area harvested for sesame seeds. The crop is mainly grown by small-holder farmers, while some commercial farmers are in Upper Nile State. In 2021, the total sesame production in South Sudan was 26,000 MT, and the yield was 0.3 tons/ha. Local demand for processed sesame seeds and byproducts, most of which are imported from neighboring countries, is growing. In 2021, South Sudan exported USD 253k in sesame oil or fractions not chemically modified, making it the 44th largest exporter in the world. Sesame is the 16th most exported product in South Sudan, the main destination of exports being the United Arab Emirates and France [7,8,10]. Sesame is regarded as one of China’s four most important traditional edible oil crops, along with soybean, peanut, and rape. Approximately 45% of sesame in China is allocated for producing sesame oil, while 22% is utilized for sesame paste, another 22% for sesame peeling, and a mere 5% for baked goods [11,12]. Sesame has long been a favorite among humans as a traditional medicinal plant with rich nutritional value and taste; also, it plays a crucial part in humans’ diet as a nutrient-dense food that is widely used in the food industry as an ingredient in various food products (e.g., bread, biscuits, burgers, cakes, dressings, dishes, snacks, and edible oil) due to its high oil content, pleasant scent, and resilience to oxidation [13]. In addition to their use as a food source, sesame seeds have extensive applications within the pharmaceutical and cosmetic sectors [14]. To produce different food products, animal feed, industrial supplies, lubricants, soaps, medicinal supplies, and co-products from sesame, the primary process of raw material is acquired [15].

**Figure 1:**
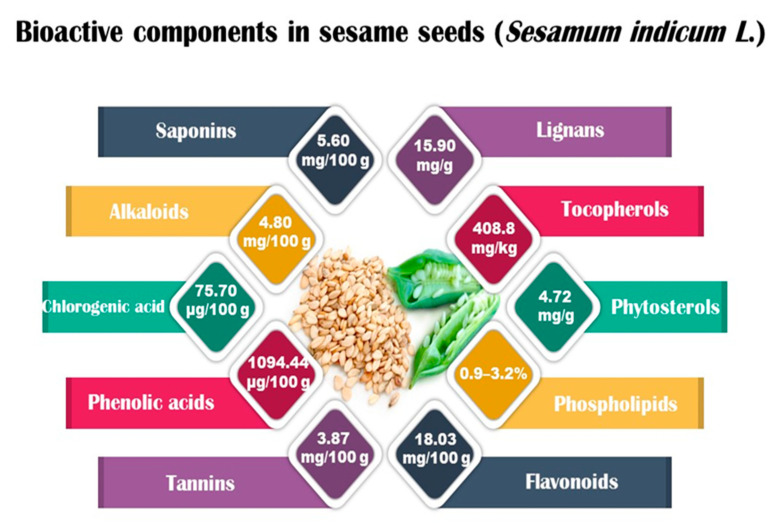
Bioactive components in sesame (*Sesamum indicum* L.).

Sesame seeds are obtainable in three distinct colors: black, brown, and white. They contain several essential nutrients in varying proportions. The composition of sesame seeds comprises 45–65% oil, a noteworthy source of plant-based protein with content ranging from 19 to 35% per 100 g of seeds, 14 to 20% carbohydrates, and 15 to 20% hull material. Although the protein content of sesame seeds is lower than that of meat, it is comparable to or higher than many grains, such as rice or wheat [16,17]. These tiny seeds also contain measurable amounts of oxalic acid, dietary fiber, antioxidants, and minerals (iron, magnesium, and zinc). The fatty acid content in sesame seeds is predominantly unsaturated fatty acids, such as oleic and linoleic acids, with smaller amounts of saturated fatty acids, like palmitic and stearic acids. Sesame oil consists of unsaponifiable fractions such as sesamin, sesamolin, and sterols. Additionally, sesame seeds are an excellent source of calcium, containing essential amino acids like methionine, valine, and tryptophan. Sesame seeds also contain bioactive components like phenolics, vitamins, phytosterols, and polyunsaturated fatty acids (PUFAs), which benefit human health [18,19]. Figure 1 shows bioactive compounds that could be detected in sesame.

Sesame lignans may account for the seed’s popularity, including sesamin, sesamolin, and sesamol. Sesame oil has been shown to have antioxidant and health-promoting benefits due to its high concentration of tocopherol, phytosterol, lignan, and other components [20,21]. The protective effects of sesame on heart function, regulation of lipid metabolism, and prevention of mutations and cancer have been demonstrated in numerous studies [22,23]. According to Ahmad and Ghosh’s (2020) research, sesame seeds possess a high nutrient content that may benefit the immune system significantly and potentially mitigate the risk of health complications associated with COVID-19. They have reported that regular consumption of sesame may lessen the likelihood of contracting viruses and stave against health issues like malnutrition that might arise [24]. Hsu and Parthasarathy [25] have indicated that the consumption of sesame oil can reduce levels of low-density lipoprotein (LDL) and decrease the risk of atherosclerosis and cardiovascular diseases. Alzheimer’s disease is linked to the deposition of toxic cellular amyloid proteins, and the prolonged consumption of sesamol may efficiently hinder this buildup [26]. 

This review presents a comprehensive overview of the structure of various bioactive compounds found in sesame seeds and products. This review also covers recent advancements in studying the impact of food processing on sesame seed oil and the mechanisms by which these technologies affect the bioactivity of sesame compounds.

## 2. The Bioactive Compounds and Health Benefits of Sesame

**Figure 2:**
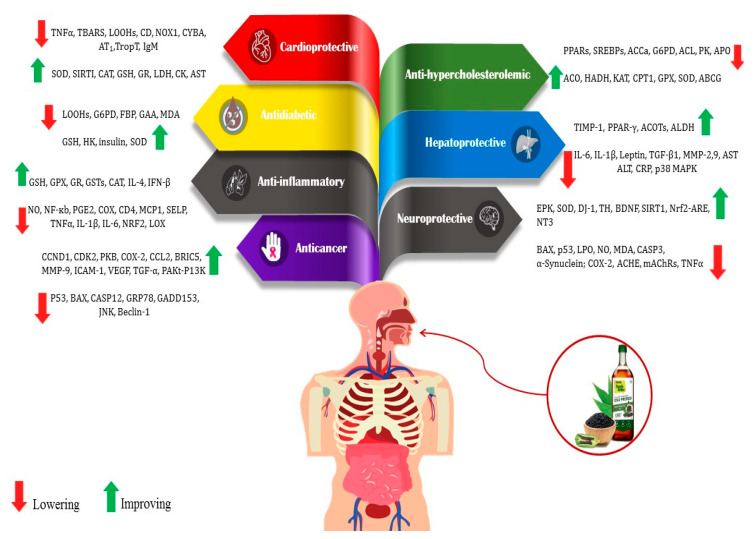
Health benefits of bioactive compounds in sesame (*Sesamum indicum* L.) and its mechanism. ABCG: ATP-binding cassette, subfamily G; ACCa: acetyl-CoA carboxylase 1; ACHE: acetylcholinesterase; ACL: ATP citrate lyase; ACO: 1-aminocyclopropane-1-carboxylic acid oxidase; ACOTs: acyl-CoA thioesterases; ALDH: aldehyde dehydrogenases; ALT: alanine aminotransferase; APOA4: apolipoprotein; AST: aspartate aminotransferase; AT1: angiotensin type 1; BAX: apoptosis regulator; BDNF: brain-derived neurotrophic factor; BIRC5: survivin protein; CASP3: caspase 3; CASP12: caspase 12; CAT: catalase; CCL2: chemokine ligand 2; CCND1: cyclin D1; CD: conjugated dienes; CDK2: cyclin-dependent kinase 2; CD4: cluster of differentiation; CK: creatine kinase; CPT1: carnitine palmitoyl transferase I; COX: cyclooxygenase; CRP: C-reactive protein; CYBA: human neutrophil cytochrome b light chain; DJ-1: protein deglycase; ERK: extracellular signal-regulated kinase; FBP: fructose 1,6-bisphosphatase; GAA: alpha glucosidase; GADD153: DNA damage-inducible gene 153; GPx: glutathione peroxidase; GR: glutathione reductase; GRP78: glucose regulatory protein 78; GSH: reduced glutathione; GSTs: glutathione-S-transferases; G6P: glucose 6-phosphate; HADH: 3-hydroxy acyl-CoA dehydrogenase; HK: hexokinase; ICAM-1: Intercellular adhesion molecule-1; IFN-β: Interferon-β; IgM: anticardiolipin antibody; IL-1β: interleukin-1β; IL-6: interleukin 6; IL-4: interleukin-4; JNK: c-Jun N-terminal kinase; KAT: 3-ketoacyl-CoA thiolase; LDH: lactate dehydrogenase; LOOHs: lipid hydroperoxide; LOX: lipoxygenase; LPO: lipid peroxidation; mAChRs: muscarinic acetylcholine receptors; MCP1: monocyte chemoattractant protein-1; MMP2: metalloproteinase-2; MMP-9: matrix metallopeptidase 9; NF-κB: nuclear factor kappa B; NO: nitric oxide; NOX1: NADPH oxidase 1; NRF2: nuclear-factor-erythroid-2-related factor 2; Nrf2-ARE pathway: transcription factor Nrf2- antioxidant responsive element; NT3: neurotrophin-3; p38 MAPK: p38 mitogen-activated protein kinases; P53: yumor protein P53; PAkt-PI3K: phosphatidylinositol 3-kinase (PI3K)/protein kinase B (AKT) signaling pathway;PGE2: prostaglandin E2; PK: pyruvate kinase; PKB: protein kinase B; PPARs: peroxisome proliferate activated receptor α; PPAR-γ: peroxisome proliferator-activated receptor gamma; SELP: P-selectin; SIRT1: sirtuin 1; SREBPs: sterol regulatory element binding proteins; TBARS: thiobarbituric acid reactive substances; TH: tyrosine hydroxylase; TGF-β1: transforming growth factor beta 1; TIMP-1: tissue inhibitor of metalloproteinases-1; TropT: troponin T;TNFα: tumor necrosis factor α; VEGF: vascular endothelial growth factor [20].

Bioactive compounds present in food are considered advantageous constituents that contribute to the prevention of diseases (Figure 2). The compounds in this category are diverse and consist of carotenoids, phenolics, phytosterols, and PUFAs [20,27,28]. The mentioned compounds are frequently employed as antioxidants and for various other functions, including but not limited to impeding cholesterol absorption and obstructing the activity of bacterial toxins [29,30,31]. PUFAs, lignans, tocopherols, and phytosterols are among the antioxidants and bioactive compounds that could be present in high levels of sesame. Compared to other edible oils, the high antioxidant content of sesame oil improves energy and resistance to aging [12,30]. 

Secondary metabolites known as natural phenolic compounds are extensively found throughout the plant kingdom. Phenolic compounds found in plants have been recognized for their potential to mitigate oxidative-stress-related conditions, including but not limited to cardiovascular and neurodegenerative disorders, as well as cancer [32,33,34,35,36,37]. Phenolics are distinguished by an aromatic ring, typically with one or more hydroxyl groups attached. Phenolics exhibit a notable antioxidative capacity owing to their ability to generate stable radical intermediates through electron utilization. Owing to their capacity to act as antioxidants, they assume a significant function in stabilizing edible oils and safeguarding against the development of undesirable flavors [38,39,40,41,42]. Recent research indicates that phenolic compounds may significantly impact ailments related to oxidative stress. The antioxidant and diverse benefits associated with sesame seed and its oil are due to the existence of lignans, specifically sesamin, sesamolin, sesaminol, sesangolin, 2-episalatin, and tocopherol isomers [18,43]. The high resistance of sesame oil to oxidative rancidity can be attributed to the chemical constituents sesamol and sesamol dimer. 

### 2.1. Lignans 

Natural products are based on the fundamental structure of a C_6_C_3_ unit, which is identified as a phenylpropanoid skeleton or propylbenzene [29,44]. The first proposal to the categorization of compounds formed by the linkage of two C_6_C_3_ units with a β,β′ bond (8-8′ bond) as lignans was proposed by Haworth [45] in a study on natural resins. Lignan, derived from two p-hydroxyphenylpropane molecules, is a constituent of lignin, a substance with a general term. Sesame seeds contain two primary groups of lignans: (I) oil-soluble lignans (sesamin, sesamolin, sesaminol, sesamolinol, and pinoresinol) and (II) glycosylated water-soluble lignans (sesaminol triglucoside, pinoresinol triglucoside, sesaminol monoglucoside, pinoresinol monoglucoside, and two isomers of pinoresinol diglucoside and sesaminol diglucoside) [46,47]. They all demonstrate various biological characteristics (Table 1) [48,49,50]. 

The antimicrobial and antioxidant efficacy ranking among the three compounds is as follows: sesamolin, sesamin, and sesamol [51,52,53]. Egawa et al. [54] reported that stimulating sympathetic nerve activity by sesame lignans improves muscle blood flow. In addition, the antioxidative properties of lignans have been observed to affect multiple models of brain dysfunction and protect against age-related brain dysfunction [20,55]. Enhancing the lignan content of sesame has become a crucial objective in sesame breeding programs because of its health-promoting properties, which have made it a functional compound [48,56]. Sesamin and sesamolin are viable options for conducting quality control assessments on samples and determining their biological value [57,58,59].

**Table 1:** Structures, quantities, biological activities, and mechanisms of different lignans detected from *Sesamum indicum*.

Lignans in Sesame	Name of Component	Molecular Structure	Quantity/Amount of Raw Sesame Seeds and Sesame Oil	Biological Characteristics	Mechanism	Reference
Oil-soluble lignans	Sesamin	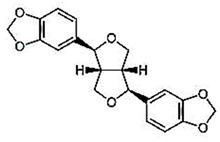 C_20_H_18_O_6_	Sesame seed: 0.77–9.3 mg/g Sesame oil: 6.20 mg/g	Antioxidant Properties	- Scavenging free radicals, such as ROS and RNS. Sesamin mitigates oxidative stress by donating electrons to these radicals, preventing cellular damage and lipid peroxidation. - Upregulating endogenous antioxidant enzymes, such as SOD, catalase, and GSH-Px. This enhancement of the cellular antioxidant defense systems contributes to the overall reduction of oxidative stress. - Through binding to transition metal ions, it engages in chelation, reducing their capacity to catalyze the formation of free radicals and thereby mitigating oxidative damage.	[50,56,60,61,62,63,64,65]
				Metabolic Health and Prevention of Diabetes	- Promoting glucose uptake into cells, thereby reducing blood glucose levels. The enhanced insulin sensitivity can contribute to the prevention of insulin resistance, a key factor in developing type 2 diabetes. - It may influence adipose tissue metabolism by modulating the expression and activity of key enzymes involved in lipogenesis and lipolysis. This regulation can contribute to maintaining a healthy balance of adipose tissue and preventing obesity-related metabolic dysregulation. - Inhibiting gluconeogenesis, the process by which the liver produces glucose. By suppressing the excessive glucose production in the liver, sesamin may prevent hyperglycemia, a characteristic feature of diabetes.	
				Cytotoxic Activity	- Inducing cell cycle arrest in different phases, such as G1, S, or G2/M, depending on the cancer cell type. This regulatory effect on the cell cycle prevents uncontrolled cell proliferation and contributes to the cytotoxic activity of sesamin. - It is associated with pharmacological activities against breast cancer. It regulates receptors such as estrogen receptor-α (ER-α), estrogen receptor-β (ER-β), and growth factor receptors (HER2 and EGFR). Also, it may suppress programmed death-ligand 1 (PD-L1) overexpression and inhibit growth factor receptors. - Sesamin interferes with these signaling cascades by modulating cell survival pathways, such as the PI3K/Akt and MAPK pathways. This disruption effectively targets the prosurvival signals crucial for the viability of cancer cells.	
				Atherosclerosis	- It promotes NO synthesis by activating endothelial NO synthase (eNOS), leading to vasodilation and improved endothelial function. - Suppresses NF-κB activation, reducing pro-inflammatory cytokines and adhesion molecules. - Interacts with PPARs, which regulate lipid metabolism and inflammation. - It may influence macrophage behavior, affecting plaque stability and regression	
	Sesamol	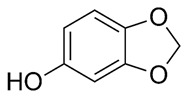 C_7_H_6_O_3_	Sesame seed: 1.20 mg/g Sesame oil: 0.27–3.37 mg/g	Cardioprotective Properties	- The molecular mechanism of cardioprotection by sesamol is primarily attributed to the methylenedioxy group present in its chemical structure. It specifically modulates the oxidative enzyme myeloperoxidase (MPO) and other proteins detrimental to human well-being.	[66,67,68,69,70,71,72,73]
				Antioxidant Activity	- The inhibition of peroxyl radicals and its pro-oxidative attack on lipid hydroperoxides and substrates. Additionally, the destructured triacylglycerol backbones influence the antioxidant activity of sesamol in fatty acid methyl esters. - Scavenging free radicals and protecting cells from oxidative damage. - It inhibits lipid peroxidation, contributing to overall cellular health. - By neutralizing ROS, it helps prevent DNA mutations and cellular dysfunction. - It is able to inhibit malic enzyme activity and NADPH supply, possibly resulting in cell proliferation and alteration in the fatty acid composition.	
				Anti-Inflammatory	- It suppresses pro-inflammatory cytokines such as IL-1β (interleukin-1β) and TNF-α (tumor necrosis factor-α). - It inhibits the NF-κB (nuclear factor kappa light chain enhancer of activated B cells) and ERK/p38 MAPK (mitogen-activated protein kinases) signaling pathways, which play crucial roles in inflammation.	
				Antiangiogenic	- It inhibits the formation of new blood vessels (angiogenesis) required for tumor growth. - By limiting blood supply to tumors, it hinders their progression.	
				Apoptosis Induction	- It promotes programmed cell death (apoptosis) in cancer cells. - It activates caspases and disrupts mitochondrial function, leading to cancer cell demise.	
				Epigenetic Regulation	- Influencing gene expression through epigenetic modifications. - It may alter DNA methylation patterns and histone acetylation, affecting cancer-related genes. - It reduces DNA damage, contributing to overall cellular integrity.	
				Detoxification and Phase II Enzyme Induction	- It can potentially increase the body’s phase II detoxification enzyme activity, assisting in removing toxic chemicals and carcinogens. The process of detoxification has the potential to prevent the development of cancer.	
	Sesamolin	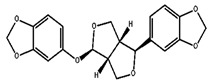 C_20_H_18_O_7_	Sesame seed: 4.50 mg/g Sesame oil: 2.45 mg/g	Neuroprotective Activity	- Reducing ROS and inhibiting apoptosis induced by hypoxia. These properties make it potentially beneficial for brain health.	[51,59,74,75,76]
				Antileukemic Effects	- Reduced leukemic cell numbers by almost 60%. - Hindered neoplastic cell proliferation. - Stimulated natural killer (NK) cell cytolytic activity against tumor cells.	
				Antimelanogenesis in Skin Cancer	- Melanin synthesis involves various enzymes; it affects the expression of melanogenic enzymes, leading to antimelanogenesis effects. - Influencing cellular signaling pathways by affecting signal transduction, it could alter the expression of genes associated with melanin synthesis.	
	Sesaminol	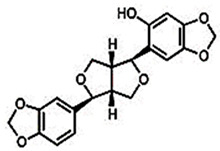 C_20_H_18_O_7_	Sesame seed: 1.40 mg/g Sesame oil: 0.01 mg/g	Anticancer Effects	- Modulating signaling pathways related to cell survival and proliferation, the induction of apoptosis (programmed cell death), and the inhibition of angiogenesis (formation of new blood vessels that support tumor growth). - Impacting key cellular signaling pathways such as the PI3K/Akt/mTOR pathway, frequently dysregulated in cancer. - Interfering with the cell cycle, preventing the uncontrolled division of cancer cells, and inducing cell cycle arrest, it restricts the ability of cancer cells to replicate and spread.	[77,78]
				Detoxification Pathways	- Activating detoxification pathways in the liver. This could play a role in protecting against carcinogens and preventing cancer initiation.	
				Preventing Parkinson’s Disease	- Activating the Nrf2-ARE signaling pathway. Nrf2 is a transcription factor that plays a key role in cellular defense mechanisms by regulating the expression of antioxidant and detoxification genes. - Activation of the Nrf2-ARE pathway by sesaminol could enhance the cellular defense against oxidative stress, which is implicated in the pathogenesis of Parkinson’s disease. - Dysfunction in mitochondrial activity; through its antioxidant and cytoprotective effects, it may contribute to the preservation of mitochondrial function in neurons.	
	Sesamolinol	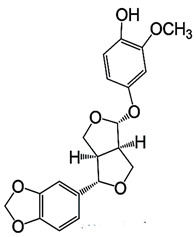 C_20_H_20_O_7_		Cardioprotective Effects	- By influencing lipid metabolism and reducing inflammation	[23,79,80]
				Hormonal Modulation	- Modulate hormone levels, particularly estrogen. This hormonal influence may have implications for conditions such as hormone-related cancers.	
				Antimicrobial Properties	- Interfering with the ability of microorganisms to adhere to host cells or surfaces. This can prevent the establishment of infections by impeding the initial steps of microbial colonization. - Some antimicrobial compounds, including certain polyphenols, exhibit chelating properties, binding to essential metal ions required for microbial growth. This can result in nutrient deprivation for the microorganisms and hinder their ability to thrive. - Inhibiting the activity of microbial enzymes, disrupting vital metabolic processes, and rendering microorganisms unable to proliferate.	
	Pinoresinol	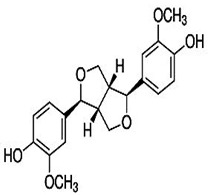 C_20_H_22_O_6_	Sesame seed: 0.29–0.47 mg/g Sesame oil: -	Hypoglycemic agent	- Inhibition of α-Glucosidase: α-Glucosidase is an enzyme that breaks down complex carbohydrates into simple sugars (glucose). Pinoresinol inhibits α-glucosidase activity in vitro. Slowing down carbohydrate digestion reduces the rate of glucose release into the bloodstream after meals. This effect helps prevent rapid spikes in blood sugar levels. - Improving insulin sensitivity in peripheral tissues (such as muscle and fat cells). Enhanced insulin sensitivity allows cells to take up glucose more efficiently, reducing hyperglycemia. - Activating AMPK, leading to increased glucose uptake by cells. AMPK activation also promotes fatty acid oxidation and overall metabolic balance.	[31,81,82,83,84,85]
				Hepatoprotective	- Down-regulates the expression of CCl_4_ hitherto and TNF-α inflammatory molecules and inhibits the production of cytotoxic cytokines by activated Kupffer cells. - Inhibiting the NF-κB and phosphorylation of c-Jun (a component of AP-1). This action suppresses the inflammatory response in liver tissue.	
				Chemoprevention	- Increasing apoptosis and cell cycle arrest.	
				Enterolignan Formation	- Pinoresinol and other plant lignans are converted into enterolignans by intestinal microflora in the human body.	
				Autophagy Induction	- Inducing autophagy, as evidenced by increased expression of LC3 II and Beclin and decreased expression of p62. Autophagy helps maintain cellular health.	
				Interaction with Gut Microbiota	- Pinoresinol, along with other plant lignans, is metabolized by intestinal microflora. These microbial transformations yield enterolignans, which may have additional health benefits. Enterolignans could influence glucose metabolism through gut–brain communication.	
Glycosylated water-soluble lignans	Sesaminol Triglucoside (STG)	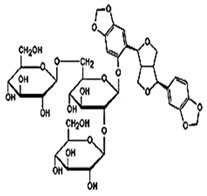 C_38_H_48_O_22_	Sesame seed: 0.36–15.60 mg/g	Glucoside Hydrolysis	- Researchers discovered a novel enzyme capable of efficiently hydrolyzing STG. This enzyme, called PSTG, is produced by a strain (KB0549) of the genus Paenibacillus. - PSTG is a tetrameric protein composed of identical subunits with an approximate molecular mass of 80 kDa. - The PSTG gene was cloned based on partial amino acid sequences of the purified enzyme. - Sequence comparison revealed that PSTG belongs to the glycoside hydrolase family 3 and shares significant similarities with Paenibacillus glucocerebrosidase and Bgl3B of Thermotoga neapolitana. - The recombinant enzyme (rPSTG) is highly specific for β-glucosidic linkage. - The kcat (catalytic rate constant) and kcat / Km (specificity constant) values for the rPSTG-catalyzed hydrolysis of p-nitrophenyl-β-glucopyraniside at 37 °C and pH 6.5 are 44 s_−1_ and 426 s_−1_ mM_−1_, respectively. - Interestingly, rPSTG exhibits higher reactivity for β-1,2-glucosidic linkage than for β-1,4- and β-1,6-glucosidic linkages. - This unique specificity allows rPSTG to efficiently decompose STG, making it the first example of such a β-glucosidase	[86,87]
	Pinoresinol Triglucoside (PTG)	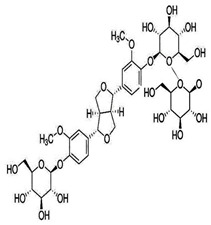 C_34_H_42_O_18_	Not explicitly documented	Autophagy Induction	PTG induces autophagy in ovarian cancer cells (SKOV-3). This is associated with increased expression of LC3 II and Beclin and decreased expression of p62.	[84,88]
				Mitochondrial Membrane Potential (MMP) Loss	It reduces the MMP of SKOV-3 cells, affecting their mitochondrial function.	
				Inhibition of Cell Invasion	It inhibits the invasion capacity of SKOV-3 cells.	
				Raf/MEK/ERK Signaling Pathway Inhibition	PTG concentration-dependently inhibits the expression of phospho-MEK and phospho-ERK, key signaling pathway components.	
				Tumor Growth Inhibition	In xenografted tumor models in mice, PTG significantly inhibits tumor growth, demonstrating its potential as an ovarian cancer treatment.	
	Sesaminol Monoglucoside (SMG)	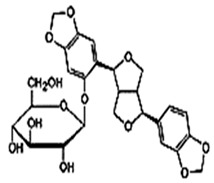 C_26_H_28_O_12_	Not explicitly documented	Antioxidant Activity	- Protecting cells from oxidative stress. - SMG undergoes enzymatic hydrolysis in the gut to release sesaminol. - Its bioavailability depends on gut microbiota, food matrix, and individual variations.	[5,89]
				Anti-Inflammatory Effects	Modulating inflammatory pathways by inhibiting pro-inflammatory cytokines.	
				Cardiovascular Health	Contributing to cardiovascular health by reducing oxidative damage and inflammation.	
				Metabolic Regulation	It is impacting lipid metabolism and glucose homeostasis.	
				Cancer Prevention	Some studies suggest that SMG may have anticancer potential, although further research is needed.	
				Cell Signaling	Influencing cell signaling pathways related to cell growth and differentiation.	
	Pinoresinol Monoglucoside (PMG)	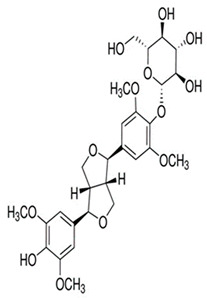 C_26_H_32_O_11_	Not explicitly documented	Metabolism and Bioavailability	PMG would undergo metabolic processes, potentially in the digestive system or the liver, leading to the release of pinoresinol and glucose. The bioavailability of pinoresinol and its metabolites would influence their distribution and effects in the body.	[48,90]
				Antioxidant Effects	scavenging free radicals and reducing oxidative stress in cells.	
				Impact on Lipid Metabolism	Modulating cholesterol levels and the promotion of cardiovascular health.	
	Pinoresinol Diglucoside (PDG)	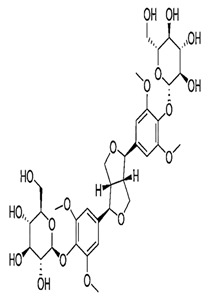 C_32_H_42_O_16_	Sesame seed: <5 to 232 mg/100 g Sesame oil: Not explicitly documented	Glucoside Hydrolysis	The glucoside structure of PDG may be hydrolyzed in vivo, leading to the release of pinoresinol. Enzymes often mediate this process, and the liberated pinoresinol can exert its biological effects.	[5,47,80]
				Antioxidant and Cytoprotective Effects	- PDG can directly neutralize free radicals by donating electrons. Free radicals are highly reactive molecules that can cause damage by stealing electrons from cellular components. By acting as electron donors, antioxidants like PDG help stabilize free radicals, preventing them from causing cellular damage. - Stimulating the activity of endogenous antioxidant enzymes within cells. These enzymes, such as SOD, catalase, and glutathione peroxidase, work to neutralize ROS and maintain cellular redox balance. - Preserving mitochondrial function, reducing the likelihood of further ROS production.	
				Impact on Lipid Metabolism	- Enhancing insulin sensitivity, potentially benefiting lipid metabolism. - Modulating inflammatory pathways and indirectly affecting lipid metabolism.	
	Sesaminol Diglucoside (SDG)	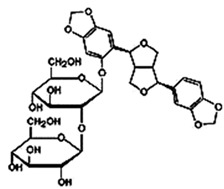 C_32_H_38_O_17_	Sesame seeds: 98 mg/100 g Not explicitly documented	Anti-Inflammatory Properties	- Modulating the production of pro-inflammatory mediators, such as cytokines (e.g., interleukins, tumor necrosis factor-alpha) and prostaglandins. - Inhibiting enzymes involved in the inflammatory process, such as COX-2 and lipoxygenase (LOX). These enzymes are responsible for synthesizing inflammatory mediators, and their inhibition can contribute to anti-inflammatory effects. - Interfering with the NF-κB signaling pathway, suppressing the transcription of pro-inflammatory genes.	[5,41,91]
				Cellular Signaling Pathways	- Influencing estrogen receptor signaling pathways, potentially impacting cellular processes associated with estrogen signaling. - Interacting with components of apoptotic signaling pathways, leading to either the promotion or inhibition of apoptosis depending on the cellular context. - Modulating MAPK/ERK pathway in regulating cell growth and differentiation.	

#### 2.1.1. Sesamol

Sesamol is a nutritional constituent and degradation byproduct of lignan derived from sesame. Identifying a novel antioxidative substance in sesame oil has highlighted its significance as a quality stabilizer and crucial aroma component [21,66,92]. Sesamol is a very-low-molecular-weight compound that is sufficiently volatile to be removed by deodorization. Consequently, it should be added to the fat after deodorization [93]. Sesamol has been documented as having various applications, including antioxidant, lipid-lowering, antidepressant, and therapeutic effects for diabetic nephropathy and neuropathy, among others [67,68]. Moreover, it has been extensively researched as an anti-inflammatory, antiatherosclerotic, and cardioprotective agent. Another research investigation has examined the potential of sesamol to enhance cognitive function and mitigate inflammation in elderly mice. The research has discovered that sesamol exhibited anti-inflammatory properties and diminished oxidative stress markers in mice, resulting in improved cognitive performance in diverse behavioral assessments. The results have indicated that sesamol could serve as a safeguard against mental deterioration and inflammatory processes associated with aging [66,69]. The study by Liu et al. [94] investigated the effects of sesamol on cognitive function in a mouse model with a high-fat- and high-fructose-diet-induced cognitive defect. They evaluated the potential of sesamol in ameliorating cognitive defects induced by the diet and investigated the insulin signaling pathways in the central nervous system. Sesamol administration significantly improved cognitive function, as confirmed by enhanced performance in the Morris water maze and novel object recognition tests. Sesamol also restored insulin signaling pathway activity in the central nervous system by increasing the expression of key proteins involved in insulin signaling. Sesamol can mitigate the production of amyloid and cognitive impairment caused by systemic inflammation by preventing neuronal injury. According to their study, sesamol has the potential to serve as a viable nutritional supplement for the prevention and treatment of obesity [94,95]. Chu et al. [70] conducted a study to investigate the potential protective effect of sesamol on endotoxemia-induced lung inflammation and injury in rats. They administered sesamol to rats and assessed its impact on endotoxemia-induced lung injury. Results indicated that sesamol administration significantly attenuated lung inflammation and injury, as demonstrated by reduced lung wet-to-dry weight ratio, decreased levels of proinflammatory cytokines, and reduced leukocyte infiltration in lung tissue. Sesamol was also found to inhibit the activation of nuclear factor-kappa B (NF-κB) and its downstream inflammatory mediators, including inducible nitric oxide synthase (iNOS) and cyclooxygenase-2 (COX-2). These findings have suggested that sesamol has the potential to protect against endotoxemia-induced lung inflammation and injury in rats, potentially through its inhibitory effect on NF-κB activation and downstream inflammatory mediators. According to Thushara et al. [96], sesamol can effectively impede platelet aggregation, exhibiting antithrombotic and cardioprotective properties. Wang et al. [97] conducted a study to explore the potential of sesamol in preventing atherosclerosis that is mediated by renal injury. According to the research, sesamol could mitigate oxidative stress and inflammation, resulting in a decline in atherosclerosis among mice suffering from renal impairment. The inhibitory effect of Sesamol on IKKα and p53 activation, both implicated in atherosclerosis pathogenesis, has been reported. The results have indicated that sesamol could potentially serve as a therapeutic agent for preventing or treating atherosclerosis linked to renal damage [98]. The research has offered a potential mechanism through which sesamol can exert its protective impact, thereby providing a basis for further investigation into the development of focused treatments for atherosclerosis. 

Chronic nephritis induces an irreversible decline in the glomerular filtration rate and the development of renal fibrosis, ultimately resulting in chronic kidney disease (CKD) and end-stage renal disease (ESRD) [99,100,101]. The infiltration of glomerular and interstitial macrophages is a hallmark of CKD, playing a pivotal role in renal injury [101,102]. Following kidney injury, damaged cells release cytokines or chemokines that recruit monocytes to inflammatory lesions, where they undergo activation and differentiation into macrophages [103,104]. Macrophages, functioning as crucial immunological regulators and inflammation mediators, are implicated in kidney damage and inflammation by secretion of inflammatory cytokines, such as interleukin (IL)-1 and IL-6 [71,105,106,107]. On-site activation and differentiation of inflammatory macrophages result in the release of IL-1, thereby triggering immune responses from Th1-type cells that contribute to tissue damage [108,109,110]. In CKD, immune cells infiltrating the kidneys play a deleterious role, actively participating in the progression of the disease and leading to nephron loss and fibrosis [111,112,113]. Tseng et al. [114] investigated the potential therapeutic effects of sesamol in addressing renal inflammation and reactive oxygen species (ROS)-mediated interleukin-1 beta (IL-1β) secretion. The study, in vivo and in vitro, focuses on the role of heme oxygenase-1 (HO-1) in inhibiting the IKKα/NFκB pathway. The findings indicated a protective role of sesamol, which revealed mitigation of renal inflammation and suppression of IL-1β secretion. The proposed mechanism involved the activation of HO-1, leading to the inhibition of the IKKα/NFκB pathway, recognized for its involvement in inflammatory processes. The study suggests the potential of sesamol as a therapeutic agent for addressing inflammatory conditions associated with renal disorders [114].

Sallam et al. [30] investigated the impact of incorporating sesame oil and sesamol as natural antimicrobial and antioxidant agents on the safety and shelf-life of meatballs. The findings of their research indicated that the incorporation of sesame oil and sesamol into meatballs resulted in a noteworthy antimicrobial impact on both Gram-positive and Gram-negative bacteria, encompassing Staphylococcus aureus, Escherichia coli, and Salmonella typhimurium. Moreover, the incorporation of sesame oil and sesamol enhanced the antioxidant potential of meatballs through the mitigation of lipid oxidation and elevation of the concentration of overall phenolic compounds. Furthermore, the sensory analysis conducted on meatballs incorporating sesame oil and sesamol indicated no statistically significant variation in flavor, appearance, and consistency compared to the control samples. The study’s results have suggested that sesame oil and sesamol possess the potential as natural preservatives for enhancing the safety and shelf-life of meat products due to their antimicrobial and antioxidant properties.

#### 2.1.2. Sesamin

Sesamin is the most prominent lignan compound found in sesame seeds, one of the two highest sources of lignans (the other being flax) in the human diet. Sesamin is catered to be a nutritional supplement that is believed to possess various properties such as anti-inflammatory, pro-apoptotic, pro-angiogenic, antimetastatic, antiproliferative, and ant-oxidant effects, as well as pro-antiphagocytic activities (if touting its health properties) or possibly being an estrogen receptor modulator and fat burner (if targeting athletes or persons wishing to lose weight) [56,60]. Sesamin has several processes, which, when considered comprehensively, may be succinctly described as a modulator of fatty acid metabolism. It seems to hinder an enzyme called Δ5-desaturase, a key enzyme in the metabolism of fatty acids. By inhibiting this enzyme, it leads to decreased levels of eicosapentaenoic acid (EPA) and arachidonic acid, two types of fatty acids found in fish oil. This effect is observed when the substance is taken orally. Sesamin inhibits the process of tocopherol-ω-hydroxylation, which is the step that limits the metabolism of vitamin E. By inhibiting this enzyme, sesamin increases the levels of vitamin E in the body, especially the γ subset (γ-tocopherol and γ-tocotrienol). This mechanism has been confirmed to be active when sesamin is taken orally [61,115,116,117].

Although there are some potentially beneficial mechanisms at play, such as protection against Parkinson’s disease and promotion of bone mass, most of the mechanisms, including estrogen receptor modulation, fat burning from the liver, and activation of the antioxidant response element (ARE), have not been verified in humans. There are reasons to doubt their occurrence, such as the possibility of the concentration being too high to have an impact through oral supplementation or the fact that fat burning appears limited to rats. Ultimately, sesamin significantly enhances the metabolism of γ-tocopherol and γ-tocotrienol by inhibiting their degradation. This leads to increased levels of these vitamin E variants, which offer numerous therapeutic advantages. Given that these vitamin E supplements are costly, sesamin could serve as a cost-effective alternative or a means to dilute the vitamin E [62,118,119,120,121].

Episesamin, an isomer of sesamin, is produced during the refining of sesame oil. Additionally, it exerts a protective influence on the levels of lipid oxidation, blood glucose, and blood pressure [62,118,122]. According to Watanabe et al. [123], the consumption of sesamin is associated with reduced blood pressure. The impact of pure sesamin epimer on serum lipids was investigated by Peñalvo et al. [124]. The study findings demonstrated that administering stanol ester alone or combined with sesamin effectively mitigated the increase in cholesterol levels. The potential has been observed in sesamin as a phytochemical agent capable of effectively impeding the differentiation and function of osteoclasts [63]. Sesamin has been documented as a protective agent against acute lung injury induced by LPS. Research has investigated the potential of sesamin to suppress microglial activation induced by LPS. According to research findings, sesamin has demonstrated the ability to decrease the expression of TLR4, a receptor that plays a role in microglia activation. This results in decreased production of pro-inflammatory cytokines and nitric oxide. The results indicate that sesamin could potentially serve as a therapeutic agent for preventing or treating neuroinflammatory conditions linked to microglial activation. The research elucidates the mechanism through which sesamin confers its protective effect, thereby providing a potential direction for further investigation in developing precise therapeutic interventions for neuroinflammatory disorders [119,125]. Zhang et al. [126] found that sesamin administration in animal models and humans was associated with a reduction in serum levels of total cholesterol, low-density lipoprotein cholesterol (LDL-C), and triglycerides, as well as an increase in high-density lipoprotein cholesterol (HDL-C). Moreover, sesamin was observed to inhibit the activity of key enzymes involved in cholesterol synthesis and fatty acid metabolism, such as 3-hydroxy-3-methylglutaryl-CoA reductase (HMG-CoA reductase) and acetyl-CoA carboxylase (ACC). Zhang et al. [127] conducted a study to examine the potential beneficial effects of sesamin on high-fat-diet-induced dyslipidemia and kidney injury in rats. They investigated the role of oxidative stress in developing these conditions and assessed the impact of sesamin on oxidative stress markers. The results showed that rats fed a high-fat diet exhibited significant dyslipidemia, characterized by elevated levels of total cholesterol, triglycerides, and low-density lipoprotein cholesterol (LDL-C), as well as reduced levels of high-density lipoprotein cholesterol (HDL-C). In addition, these rats exhibited kidney injury, as evidenced by increased serum creatinine levels and blood urea nitrogen (BUN). However, supplementation with sesamin at both low and high doses was observed to ameliorate these effects. They have found that sesamin administration significantly reduced serum levels of total cholesterol, triglycerides, and LDL-C, as well as increased HDL-C. Furthermore, sesamin was found to decrease oxidative stress markers, including malondialdehyde (MDA) and reactive oxygen species (ROS), and increase the activity of antioxidant enzymes, such as superoxide dismutase (SOD) and glutathione peroxidase (GSH-Px). In another study, the results showed that sesamin increased the expression of key proteins involved in cholesterol efflux, such as ATP-binding cassette transporter A1 (ABCA1) and scavenger receptor class B type I (SR-BI), while decreasing the expression of proteins involved in cholesterol uptake, such as CD36 and scavenger receptor class A (SR-A) [128]. A study was conducted in HepG2 cells, a human liver cell line, and used various techniques to determine the effect of sesamin on lipid metabolism and gene expression. The results showed that sesamin inhibited lipid accumulation in HepG2 cells by downregulating the expression of key lipogenic genes, such as sterol regulatory element-binding protein 1c (SREBP-1c) and fatty acid synthase (FAS), through interaction with LXRα and PXR. The findings suggest that sesamin has the potential to be used as a therapeutic agent for preventing or treating lipogenesis-associated diseases such as steatosis [129]. Various studies have recognized Sesamin as a potent agent with promising immunomodulatory and anti-inflammatory effects. It has been shown to have the potential to modulate the immune response by regulating the production of cytokines, which are key players in the inflammatory response. Sesamin has been observed to inhibit the production of pro-inflammatory cytokines, such as interleukin-1β (IL-1β), interleukin-6 (IL-6), and tumor necrosis factor-alpha (TNF-α), while promoting the production of anti-inflammatory cytokines, such as interleukin-10 (IL-10). This suggests that sesamin may have therapeutic potential in treating various inflammatory disorders. Moreover, sesamin has been reported to have potent antioxidant properties, which may contribute to its anti-inflammatory effects. The ability of sesamin to reduce oxidative stress and inflammation may make it a potential therapeutic candidate for conditions associated with chronic inflammation, such as cardiovascular disease, cancer, and neurodegenerative disorders [118]. Sesamin is effective as an adjuvant therapeutic agent in treating cardiovascular diseases (CVD) [62]. The anticancer potential of sesamin against non-small cell lung cancer (NSCLC) cells has been investigated by Chen et al. [130]. According to the study, sesamin has demonstrated the ability to impede the proliferation of NSCLC cells and trigger apoptosis, or programmed cell death, through the Akt/p53 pathway. The researchers employed various experimental techniques, including the MTT assay, flow cytometry, Western blotting, and immunofluorescence staining, to examine the mechanisms underlying the impact of sesamin on NSCLC cells. The results indicated that sesamin exhibits promise as a viable therapeutic intervention for treating non-small cell lung cancer. In summary, the research has presented compelling data regarding the potential antineoplastic effects of sesamin, indicating the need for additional exploration of this substance in the context of novel cancer treatments. Sesamin exhibited a significant inhibitory effect on tumor growth in vivo. 

#### 2.1.3. Sesamolin

Sesamolin is a well-known lignan in sesame oil that exhibits noteworthy antimutagenic, antiaging, and antioxidant characteristics. The results of a study by Nagarajan and Lee [74] have suggested that sesamolin could be a promising antileukemic agent in vivo based on its efficacy in a wehi-3B-induced leukemia model. According to Tsai et al. [131] research, it has been demonstrated that the antioxidant activity of related derivatives of sesame lignans may surpass that of endogenous lignans present in sesame oil. In another research, the effectiveness of sesamolin in reducing serum and liver lipid levels, along with a simultaneous increase in liver fatty acid oxidation, was observed [132]. Yu et al. [133] conducted a study investigating the therapeutic effects of sesamolin on nonalcoholic fatty liver disease (NAFLD) in mice fed a diet high in fat and fructose. The research aimed to investigate sesamolin’s capacity to regulate the gut microbiota and metabolites in individuals with NAFLD. The study involved the partitioning of 40 C57BL/6J mice into four distinct groups, namely, normal chow diet (NCD), high-fat and high-fructose diet (HFD), HFD with low-dose sesamolin (HFD+L-SES), and HFD with high-dose sesamolin (HFD+H-SES). The experimental subjects were mice that underwent a 16-week treatment regimen involving sesamolin. The study findings indicate that administering sesamolin resulted in a decrease in body weight, liver weight, and serum levels of alanine transaminase (ALT) and aspartate transaminase (AST) in the HFD+H-SES group as compared to the HFD group. Sesamolin exhibited a mitigating effect on hepatic lipid accumulation and inflammation induced by a high-fat diet. Moreover, the examination of gut microbiota demonstrated that administering sesamolin augmented the prevalence of advantageous bacteria, such as Akkermansia, and reduced the prevalence of detrimental bacteria, such as Desulfovibrio. According to the results of the metabolic analysis, the administration of sesamolin resulted in an elevation of short-chain fatty acids (SCFAs), bile acids, and amino acids, alongside a decrease in branched-chain amino acids (BCAAs) and lipids. The research findings suggest that administering sesamolin may have a beneficial effect on NAFLD in mice subjected to a diet high in fat and fructose. This effect is believed to be achieved by regulating gut microbiota and metabolites. The research presents a novel therapeutic strategy for managing NAFLD using sesamolin as a dietary adjunct. The potential of Sesamolin as a therapeutic agent for tumors lies in its ability to regulate the differentiation and activation of dendritic cells, leading to the efficient activation of natural killer cells [134]. According to Lee and Lee’s findings [135], it was observed that the cytolytic activity of natural killer cells is stimulated by sesamolin. The objective of the research conducted by Mohamed et al. [136] was to examine the potential neuroprotective properties of sesame oil in Alzheimer’s disease (AD) and explore the molecular mechanisms that may be involved in this protective effect. The AD rat model was created by administering amyloid-β (Aβ) 1–42 through intracerebroventricular injection, a recognized feature of AD pathogenesis. The study’s findings indicate that the administration of sesame oil had a notable effect in reducing memory impairment caused by Aβ, as demonstrated by the Morris water maze test results. In addition, the administration of sesame oil demonstrated a reduction in oxidative stress within the brain, as indicated by a decrease in malondialdehyde (MDA) levels and an increase in reduced glutathione levels. (GSH). The study found that using sesame oil resulted in a neuroprotective effect linked to suppressing the nuclear factor kappa B (NF-κB)/p38 mitogen-activated protein kinase (MAPK) signaling pathway. The observed reduction in the levels of p-NF-κB and p-p38 MAPK supported this. In addition, the administration of sesame oil increased brain-derived neurotrophic factor (BDNF) and peroxisome proliferator-activated receptor gamma (PPAR-γ) levels. These neurotrophic factors play a crucial role in neuroprotection and neuronal plasticity. The study’s results suggest that sesame oil can be utilized as a therapeutic intervention for AD. This may be due to its ability to regulate the NF-κB/p38MAPK/BDNF/PPAR-γ pathways [136]. Sesamolin has the potential to effectively stop the death of primary cortical cells brought on by hypoxia [137]. Sesamolin exhibits potential as a bioactive compound in vivo and may serve as a viable therapeutic agent for various diseases [59,89]. Katayama et al. [138] have investigated the impact of sesaminol, a substance obtained from sesame seeds, on the buildup of amyloid beta (Aβ) in the brains of senescence-accelerated mouse-prone 8 (SAMP8) mice, which serve as a model for Alzheimer’s disease. The study involved administering a diet containing sesaminol or a control diet to mice for 10 weeks. The researchers subsequently assessed the levels of Aβ in the brain and the activity of enzymes responsible for its production and degradation. The findings have indicated that the sesamol regimen had a notable impact on the reduction of Aβ buildup in the murine brain, as well as a concomitant decrease in the enzymatic activity responsible for Aβ production and an increase in the enzymatic activity that facilitates its degradation. Furthermore, consuming a sesamol-enriched diet resulted in elevated levels of BDNF. This protein plays a crucial role in supporting the survival and proliferation of neurons while concurrently decreasing the levels of oxidative stress markers in the brain. The results of this study indicated that sesaminol could serve as a viable therapeutic option for the prevention or treatment of Alzheimer’s disease. This is due to its ability to decrease the accumulation of Aβ and enhance the survival of neurons. Keowkase et al. [139] conducted a study to examine the impact of sesamin and sesamolin, two primary lignans found in sesame seeds, on the toxicity of amyloid-β (Aβ) in a transgenic Caenorhabditis elegans model of Alzheimer’s disease. The investigation was carried out by evaluating the paralysis of worms, their lifespan, markers of oxidative stress, and accumulation of Aβ. The findings indicate that sesamin and sesamolin can mitigate the toxicity induced by Aβ, prolong the lifespan of nematodes, and delay paralysis. Furthermore, the two lignans exhibited a reduction in the accumulation of Aβ in transgenic nematodes and a decline in oxidative stress markers, including reactive oxygen species (ROS) and malondialdehyde (MDA) levels. The authors suggested that the observed outcomes could be attributed to sesamin and sesamolin’s antioxidative and neuroprotective characteristics. Hence, it is probable that sesamin and sesamolin possess therapeutic properties that could be employed in treating Alzheimer’s disease. Nevertheless, additional research is necessary to ascertain their precise mechanism of operation and clinical effectiveness. 

### 2.2. Tocopherols

Tocochromanols exhibit amphipathic characteristics, possessing both hydrophobic and hydrophilic components. Typically, these biomolecules comprise a lipophilic isoprenoid side chain attached to the membrane lipids and a polar chromanol ring oriented towards the membrane surface. Tocochromanols can impede the membrane lipid peroxidation process and function as scavengers of reactive oxygen species. Antioxidants have been widely recognized for their ability to counteract the harmful effects of free radicals, which helps mitigate DNA damage. Tocopherols function as scavengers of reactive oxygen species, thereby mitigating the impact of free radical attack and disrupting lipid peroxidation. These molecules safeguard cell membranes, facilitate lipid restoration and substitution, and exhibit utility in preventing cancer and cardiovascular ailments [140,141,142,143,144]. Tocopherols have been identified as having a significant function in plant metabolism, specifically in transporting sugar from leaves to phloem [145,146,147]. 

Tocopherols are a significant plant phenolic compound class that possesses both antioxidative properties and nutritional benefits. The molecules in question pertain to a family characterized by the presence of a chromanol ring, which is a type of chroman ring featuring an alcoholic hydroxyl group, as well as a 12-carbon aliphatic side chain that includes two methyl groups located centrally and two additional methyl groups situated at the terminus [148,149]. Plants can produce eight distinct forms of vitamin E, which encompass α-, β-, γ-, and δ-tocopherols and α-, β-, γ-, and δ-tocotrienols. Both tocopherols and tocotrienols comprise a chromanol ring and a variable quantity of methyl groups on the chromanol ring. Tocols have two main components: the chromanol ring and the hydrophobic side chain. Tocopherols and tocotrienols differ based on the acyl side chain they possess. The tocopherols have hydrophobic side chains that consist of saturated isoprenoid chains, whereas the tocotrienols have hydrophobic side chains that consist of isoprenyl chains with three double bonds. The chromanol ring can provide a hydrogen atom to reduce free radicals, while the hydrophobic side chain enables the molecule to permeate biological membranes [138,139] effectively. Tools’ metabolic outcomes and physiological effects are contingent upon their inherent structural characteristics. All the isoforms function as lipid antioxidants, with α-tocopherol exhibiting the greatest vitamin E activity [150,151,152,153]. Tocopherols are present in various plant organs of dicotyledonous species, encompassing roots, stems, leaves, flowers, fruits, and seeds. Nevertheless, a significant disparity exists in the overall tocopherol concentration and various tocopherol forms present in these biological tissues. The prevailing tocopherol variant in photosynthetic tissues, such as stems and leaves, is α-tocopherol. The prevalence of γ- and δ-tocopherols is typically higher than that of α-tocopherol in most seed crops [154,155,156]. The α-tocopherol content in sesame seeds varies but is usually present in trace amounts. Studies have reported levels ranging from 18.51 mg/100g to 49.63 mg/100g [157]. While sesame seeds contain other beneficial compounds, such as γ-tocopherol and δ-tocopherol, α-tocopherol is not the predominant form in seeds. The α-tocopherol content in sesame oil also varies depending on the extraction process and purity. Sesame oil may sometimes contain higher levels of α-tocopherol than the seeds. However, the exact amount can differ significantly. One study found that sesame seed oil extracts had a higher total phenolic content (TPC) than α-tocopherol. Specifically, the TPC in sesame oil was 26.00 mg GAE/g of extract, while α-tocopherol was 18.00 mg GAE/g [158].

Another study detected α-tocopherol concentrations in vegetable oils, including sesame oil, expressed as mg/kg. The values for α-tocopherol in sesame oil ranged from 71.3 ± 6.4 mg/kg to 432.3 ± 86.6 mg/kg [159]. The primary function of α-tocopherol as an antioxidant disrupts radical chains in lipoproteins and membranes. Its antioxidant potential and molecular functions help to mitigate the possibility of cardiovascular disease and cancer. Although in smaller amounts, other tools also have antioxidative and biological activity [160]. γ-tocopherol, for example, is more effective than α-tocopherol in reducing platelet aggregation, LDL oxidation, and intra-arterial thrombus formation. Tocotrienols can inhibit cholesterol biosynthesis and lower the risk of breast cancer. Tocopherols have been found to possess significant potential as antitumor agents, antioxidants, and hypocholesterolemic agents. The antiallergic properties of α-tocopherol were examined in one study using a mouse model of allergic rhinitis. α-tocopherol was administered to mice after they had been exposed to ovalbumin to create a sensitized state for the experiment. As seen by a drop in serum IgE levels and nasal symptom ratings, the findings demonstrated that α-tocopherol inhibited allergic reactions in the mice. The PI3K-PKB signaling pathway, which is essential for mast cell activation and degranulation, was shown to be blocked by α-tocopherol in in vitro tests using mouse mast cells. According to the research, α-tocopherol, a crucial receptor implicated in the allergic response, was also shown to reduce the expression of FcεRI on the surface of mast cells. The research indicated that α-tocopherol may have a therapeutic benefit for allergic rhinitis, presumably via inhibiting the mast cell PI3K-PKB signaling pathway [161,162,163,164].

Sesame seeds contain a combination of tocopherols and tocotrienols, with α-tocopherol and γ-tocopherol being the primary contributors to their beneficial properties (Table 2). 

Tocotrienols are compounds belonging to the vitamin E group. Tocotrienols are less prevalent in nature compared to other types of vitamin E. The majority of our dietary intake consists of tocopherols rather than tocotrienols. Tocotrienols bioavailability varies, and they are often found in lower concentrations in the bloodstream. Tocotrienols have a similar basic structure to tocopherols, consisting of a chromanol ring and a phytyl tail. However, tocotrienols have three double bonds in their phytyl tail, distinguishing them from tocopherols. The four main tocotrienols are α, β, γ, and δ. γ -tocotrienol is the most abundant and studied form. Tocotrienols are found in certain plant-based oils, seeds, and grains. Notable sources include palm oil, rice bran oil, barley, wheat germ, and sesame seeds. However, some vegetable oils, such as palm oil, are rich in tocotrienols. The majority of vitamin E supplements often include tocopherols rather than tocotrienols. Research also indicates that tocotrienol is a more potent source of vitamin E than tocopherol. Research shows that tocotrienol has several health advantages [140,150]. Tocotrienols are known for their unique antioxidant properties that hinder plasma cholesterol levels and are linked with the prevention of cardiovascular disease [165]. Tocopherols were found to have the potential to mitigate stress-induced pathological alterations [166,167]. Dietary sesame seeds have been shown to increase both α-tocotrienol and γ-tocotrienol concentrations in specific tissues of rats. The research found that rats with a diet containing sesame seeds observed higher amounts of tocotrienols in their adipose tissue and skin. Nevertheless, these effects were not seen in plasma or any other tissue in the body. Remarkably, sesame seeds also increased the level of γ-tocopherol in different tissues despite its initial scarcity [168,169]. The combined application of tocotrienol and sesame lignans has been observed to exhibit a preventive effect against oxidative damage caused by UVB irradiation [170]. The tocopherol concentration in sesame oil ranges from 530 mg/kg to 1000 mg/kg. The predominant form of tocopherol found in sesame oil is γ-tocopherol, with concentrations ranging from 521 to 990 mg/kg. Additional tocopherols include δ (4 mg to 20 mg/kg) and α (up to 3 mg /kg). In addition, sesame oil may consist of a maximum of 20 mg /kg of γ-tocotrienol [157,171,172]. Morris et al. [173] have suggested that functional health foods can benefit from using δ- and γ-tocopherols within the concentration range of 214 to 239 μg/g. In addition to tocopherols and lignans, sesame has been found to contain small amounts of phenolic acids and naphthoquinone, which are also phenolic compounds with potential health benefits [5].

**Table 2:** Structures, quantities, biological activities, and mechanisms of different tools detected from *Sesamum indicum*.

Tocols in Sesame	Name of Component	Molecular Structure	Quantity/Amount of Raw Sesame Seeds and Sesame Oil	Biological Characteristics	Mechanism	Reference
Tocopherols	α-Tocopherol	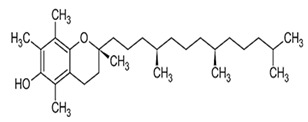 C_29_H_50_O_2_	Sesame seeds: 18.51–49.63 mg/100 g Sesame oil: 71.3 ± 6.4–432.3 ± 86.6 mg/kg	Antioxidant Action	- Act as lipophilic antioxidants. - It scavenges lipid peroxy radicals and quenchs singlet oxygen. - The antioxidant action involves the formation of tocopherol quinone. - It plays a crucial role in protecting photosynthetic organisms by combating oxidative stress and maintaining cellular health12.	[157,158,159,174]
	γ-Tocopherol	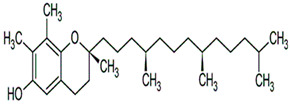 C_28_H_48_O_2_	Sesame seeds: 169–577 mg/kg Sesmae oil: 329–1114 mg/L	Antioxidant properties	Guards against lipid oxidation.	[175]
				Potential Cancer Prevention	Maintains cell integrity	
				Immune Support	Modulates gene expression	
	δ-Tocopherol	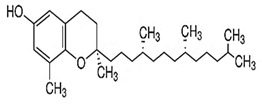 C_27_H_46_O_2_	Sesame seeds: 0.1–1.5 mg/100 g Sesame oil: 5–10 mg/100 g	Antioxidant Properties	- It scavenges ROS and prevents lipid peroxidation, maintaining the quality of sesame oil. - Modulating gene expression related to inflammation and cell survival.	[29,167]
				Health benefits	- Cardiovascular health: δ-Tocopherol contributes to heart health by reducing oxidative stress and inflammation. - Cancer prevention: its antioxidant properties may help prevent cancer development. - Immune system support: it supports immune function by neutralizing harmful radicals.	
Tocotrienols	α-Tocotrienol	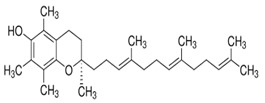 C_29_H_44_O_2_	Sesame seeds: 0.134 mg/100 g Sesame oil: Not detected	Antioxidant Properties	- It is a potent fat-soluble antioxidant that inhibits lipid peroxidation in biological membranes. - It has higher antioxidative activity than α-tocopherol.	[169]
				Gene Modulation	- Inhibitory effects on cell growth and differentiation in tumor cell lines. - Supplementation with tocotrienol-rich fractions affects genes involved in cell cycle regulation and tumor cell growth.	
	γ-Tocotrienol	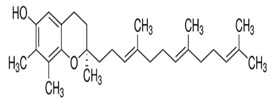 C_28_H_42_O_2_	Sesame seeds: 0.415 mg/100 g Sesame oil: Up to 20 mg/kg	Specific Properties	- Unlike α-tocopherol, γ-tocotrienol exhibits distinct biological activities that are not solely related to its antioxidant capacity. It binds to ERβ, which may contribute to its unique effects.	[157]
				Neuroprotection	- It protects cells from oxidative damage caused by free radicals.	

### 2.3. Phytosterols

Phytosterols, which encompass sterols and stanols, are triterpenes derived from plants. They serve as crucial constituents of cell membranes and exhibit preventive properties against various diseases, notably cancer. Research has demonstrated that these entities exhibit characteristics of being antioxidants and anti-inflammatory and antibacterial agents [176,177,178,179]. Phytosterols, possessing an additional methyl group at the C-24 position, show a structural resemblance to cholesterol. Upon digestion, these plant sterols effectively compete with cholesterol for absorption in the small intestine, thereby reducing cholesterol levels in the bloodstream. Functional foods commonly recommended for their cholesterol-lowering properties often incorporate phytosterols derived from plant sources. Devaraj and Jialal [180], as well as Weingärtner et al. [168], have suggested that the incorporation of phytosterols in functional foods has been employed as a therapeutic measure for hypercholesterolemia and the mitigation of plasma cholesterol concentrations. Numerous studies have indicated that phytosterols can reduce blood cholesterol levels, enhance immune system function, and lower the incidence of particular cancers [181,182,183,184]. Alternatively, processed foods may be fortified with phytosterols and marketed as supplements for cholesterol management [185,186,187]. Phytosterols have the potential to serve as health-promoting constituents that may mitigate low-density lipoprotein and cholesterol levels, thereby averting the onset of cardiovascular disease [188,189]. Phytosterols have been shown to provide protection against prostate, breast, and colon cancer, as demonstrated in previous studies [190,191,192]. Meanwhile, Llop-Talaveron et al. [193] have recently published findings suggesting that phytosterolemia may protect against liver complications commonly associated with parenteral nutrition. In contrast, Nzekoue et al. [194] have observed that phytosterol oxidation products may form phytosterol oxidation products, which possess pro-inflammatory and pro-atherogenic properties. While phytosterols can be extracted from corn and legumes, it is noteworthy that sesame seeds contain the highest concentration of phytosterols, with a range of 400–413 mg per 100 g. Table 3 shows different classes of phytosterols that could be detected in *sesamum indicum.*

Gharby et al. [195] reported that β-sitosterol is a major component of phytosterols in sesame seed and oil, accompanied by campesterol and stigmasterol. β-sitosterol is the primary phytosterol present in sesame oil. A group of researchers discovered that the level of phytosterols present in brown sesame cultivars was comparatively greater than in white sesame cultivars [196,197]. β-Sitosterol has been studied extensively and shown to have significant potential for promoting human health. It exhibits cholesterol-lowering effects, boosts immunity, and exhibits anti-inflammatory properties [198,199,200,201]. Campesterol, another major sterol found in sesame oil, constitutes approximately 17.8% of total sterols, while Δ5-avenasterol and stigmasterol are present at around 10.2% and 6.4%, respectively. The minor sterols Δ7-stigmasterol and Δ7-avenasterol are also present. Sesame seed oil contains a total sterol content of about 540 mg/100 g oil [31,202]. 

**Table 3:** Structures, quantities, biological activities, and mechanisms of different phytosterols detected from *Sesamum indicum*.

Phytosterols in Sesame	Molecular Structure	Quantity/Amount of Raw Sesame Seeds and Sesame Oil	Biological Characteristics	Mechanism	Reference
β-Sitosterol	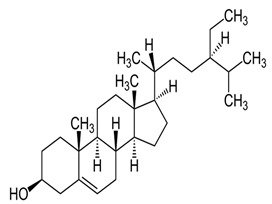 C_29_H_50_O	Sesame seed: 3.35 mg/g Sesame oil: 2.63 mg/g	Antitumor Effects	- Reducing cell proliferation by interfering with cell cycle progression. It can arrest tumor cells in specific cell cycle phases, preventing uncontrolled growth. - Suppressing tumor cell migration and invasion, crucial steps in metastasis. It modulates signaling pathways involved in cell movement and tissue invasion. - Enhancing the sensitivity of tumor cells to chemotherapy drugs. It can make cancer cells more susceptible to treatment, improving therapeutic outcomes	[29,179,203,204,205]
			Anti-Inflammatory Properties	- Reducing the production of inflammatory mediators such as IL-6, iNOS, TNF-α, and COX-2. - Modulating NF-κB signaling. - Inhibiting inflammatory pathways in adipose tissue and liver.	
			Antidiabetic	- Improves insulin sensitivity by enhancing receptor signaling pathways in target tissues (such as muscle and adipose tissue). It promotes glucose uptake and utilization. - Activates AMPK, a key regulator of energy metabolism. AMPK activation enhances glucose uptake and utilization, leading to improved glycemic control. - Competes with cholesterol for absorption in the intestine, reducing cholesterol levels. Lower cholesterol levels positively impact insulin sensitivity. - May reduce elevated triglyceride levels associated with insulin resistance.	
			Ameliorative Effects on Prostatic Hyperplasia	- It is associated with increased dihydrotestosterone (DHT) levels, a potent androgen. - Inhibiting the enzyme 5α-reductase, which converts testosterone to DHT. - Reducing DHT levels helps prevent prostate enlargement. - Supplementation improves urinary flow rate, reduces residual urine volume, and alleviates symptoms like frequent urination, nocturia, and weak stream in BPH patients.	
			Hepatoprotective	- Scavenging free radicals reduces lipid peroxidation and maintains cellular redox balance. - Normalizing liver enzymes (such as alanine aminotransferase, ALT, aspartate aminotransferase, and AST) that are elevated during liver damage. - Mitigating liver injury induced by hepatotoxic agents such as carbon tetrachloride (CCl_4_) or alcohol. - Helps normalize lipid levels in the liver by reducing excessive accumulation of triglycerides and cholesterol, preventing fatty liver disease.	
Campesterol	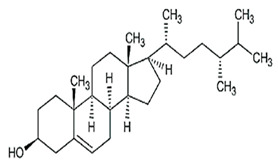 C_28_H_48_O	Sesame seed: 1.00 mg/g Sesame oil: 1.35 mg/g	Antioxidant Properties	- Scavenging free radicals. It donates electrons to stabilize these radicals, preventing them from causing cellular harm. - Reducing lipid peroxidation by neutralizing free radicals and protecting cell membranes from damage.	[29,206,207]
			Cardiovascular Health	- Decreasing LDL cholesterol levels overall. Elevated LDL cholesterol is a risk factor for cardiovascular diseases, including atherosclerosis and heart disease. - Reducing the production of pro-inflammatory cytokines (such as TNF-α and IL-6) in blood vessels and tissues. - Enhancing nitric oxide production promotes vasodilation and maintains healthy blood flow. - By maintaining cholesterol homeostasis, campesterol contributes to cardiovascular health.	
			Cholesterol Regulation	- By modulating biosynthesis, it helps prevent excessive cholesterol buildup. - It localizes to cell membranes, interacting with adjacent lipids, and helps regulate membrane rigidity, fluidity, and permeability. By binding to transmembrane proteins, campesterol can alter their conformations.	
Stigmasterol	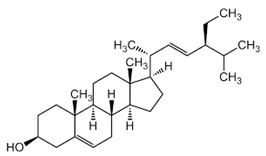 C_29_H_48_O	Sesame seed: 0.37 mg/g Sesame oil: 0.47 mg/g	Antiproliferative Activity	- Modulating cyclin proteins and cyclin-dependent kinases (CDKs).	[29,208]
			Mitochondrial Regulation and ROS Generation	- Regulating the PI3K/Akt signaling pathway. - Generating mitochondrial ROS. - Disrupting mitochondrial function, leading to cell death.	
			Autophagy Induction	- Activating AMPK, a key cellular energy sensor. AMPK activation is associated with the induction of autophagy, promoting energy homeostasis under nutrient stress. - Inhibiting the mechanistic target of the rapamycin (mTOR) pathway. - Activating extracellular signal-regulated kinase 1/2 (ERK1/2) and JNK - Inducing endoplasmic reticulum (ER) stress. ER stress can activate autophagy as a cellular adaptive response to restore ER homeostasis. - Upregulating Beclin-1 expression. Beclin-1 is a crucial autophagy-related protein involved in the nucleation of autophagosomes, promoting autophagy initiation.	
Sitostanol	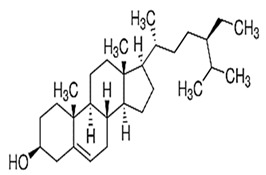 C_29_H_52_O	Sesame oil: 0.04 mg/g	Mitochondrial Respiration	- In human brown adipocytes, myotubes, and hepatocytes, sitostanol’s impact on mitochondrial function was investigated. - In HepG2 human hepatocytes, maximal mitochondrial function was decreased following sitostanol incubation, particularly when assessed in low glucose-containing medium	[29,184,209]
			Cholesterol Regulation	- Reducing cholesterol absorption from the diet by interfering with its incorporation into mixed micelles. This decreases cholesterol uptake by enterocytes and subsequently lowers circulating cholesterol levels. - Influencing cholesterol homeostasis by modulating the expression of genes involved in cholesterol synthesis, uptake, and excretion. - Enhancing the excretion of cholesterol via the feces. By reducing cholesterol absorption, unabsorbed cholesterol accumulates in the intestine, which is then excreted in the feces.	
Campestanol	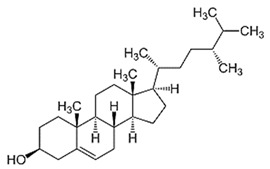 C_28_H_50_O	Sesame oil: 0.02 mg/g	Enzyme Inhibition	- Inhibiting key enzymes involved in cholesterol synthesis, particularly HMG-CoA reductase, in vitro. Inhibition of these enzymes reduces endogenous cholesterol production within cells.	[187,210]
			Antiatherogenic Effects	- Lecithin: cholesterol acyltransferase (LCAT) activity: influences cholesterol metabolism. - Bile acid synthesis: affects cholesterol homeostasis. - Oxidation and uptake of lipoproteins: impacts lipid balance. - Hepatic and lipoprotein lipase activities: regulation of lipid processing. - Coagulation system: this may contribute to overall cardiovascular health.	
∆5-avenasterol	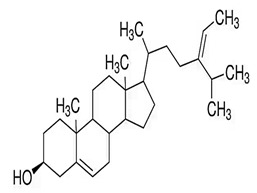 C_29_H_48_O	Sesame oil: 0.82 mg/g	Neutralization Stoichiometry	- Impact on viral entry and infectivity is typically assessed through in vitro neutralization assays. - These assays measure the ability of antibodies (including ∆5-Avenasterol) to block viral entry into target cells. - Neutralization is crucial for predicting antiviral efficacy.	[211,212]
			Antiviral Functions	- Neutralization: blocking viral entry into host cells. - Antibody effector functions: these include interactions with immune cells and complement systems. - The relative contributions of these mechanisms to ∆5-Avenasterol’s overall antiviral efficacy vary depending on the specific antibody–virus interactions.	
Δ5-Stigmasterol	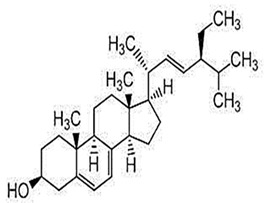 C_29_H_48_O	The specific amount was not detected	Antitumor Activity	- Regulating the PI3K/Akt signaling pathway. - Generating mitochondrial reactive oxygen species. - Modulating cyclin proteins and -CDK	[208,213]
			Neuroprotection Against Oxidative Stress	- Reducing ROS: Δ5-Stigmasterol maintains ROS levels inside cells, preventing oxidative stress-induced cell death. - Upregulation of neuroprotective proteins. - Forkhead box O (FoxO) 3a: associated with cell survival. - Catalase: an antioxidant enzyme. - B-cell lymphoma 2 (Bcl-2): antiapoptotic protein. - Activation of Sirtuin 1 (SIRT1): Δ5-Stigmasterol stimulates SIRT1 activity, similar to the known SIRT1 activator, resveratrol.	
Δ7-avenasterol	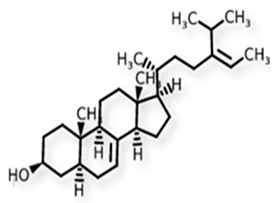 C_29_H_48_O	The specific amount was not detected	Protection Against Leishmania	- Modulating the host immune response against Leishmania. Influencing immune cells and cytokine production might contribute to an environment that is less conducive to the survival and proliferation of the parasite. - It directly interacts with the parasites, affecting their membrane integrity or interfering with essential cellular processes. - Triggering oxidative stress responses in Leishmania, leading to damage to cellular structures and compromising the viability of the parasites.	
			Improving Learning and Memory Ability	- Protecting neurons from damage and supporting their health is fundamental to maintaining cognitive function. - Modulating neurotransmitter systems. Neurotransmitters play a key role in learning and memory; any substance affecting their release or function could influence cognitive abilities. - Substances that effectively cross the blood–brain barrier (BBB) may have a more direct influence on cognitive processes.	
			Potential Antioxidant Function	- Modulating the activity of endogenous antioxidant enzymes. It may enhance the expression and activity of enzymes such as SOD, CAT, and GPx, contributing to the cellular antioxidant defense system. - Preserving endogenous antioxidants like glutathione. By maintaining the levels of these antioxidants, it reinforces the cellular defense mechanisms against oxidative stress. - Preventing an imbalance between oxidants and antioxidants, ensuring cells function optimally without succumbing to oxidative stress.	
Δ7-Stigmastenol	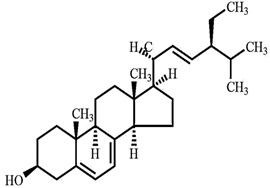 C_29_H_50_O	The specific amount was not detected	Gene Expression Analysis	- Effect on gene expression related to cholesterol metabolism is analyzed in vitro. This includes assessing its influence on the expression of enzymes involved in cholesterol biosynthesis and transport.	[197,214]
			Cholesterol Regulation	- Disrupting the transport of cholesterol in the intestine. This disruption limits the incorporation of cholesterol into chylomicrons, decreasing the transport of dietary cholesterol.	

### 2.4. Phytates 

Phytic acid is a bioactive compound commonly present in various plant-based food sources. The molecular configuration of the substance enables it to form a complex with polyvalent cations, including minerals and trace elements. Phytic acid serves as a vital reservoir of phosphorus in plant seeds, with sesame seeds exhibiting a greater concentration of phytate than legumes [215]. Oil seeds, including linseed, rapeseed, sesame, soybean, and sunflower, display a phytic acid content that varies between 1% and 5.4%, whereas legumes typically contain 0.2% and 2.9%. The phytate concentration in sesame meal is higher than in soybean meal [216]. According to Touma et al. [217], the phytic acid content in sesame seeds is 5.36%. Phytates have also been discovered to possess hypocholesterolemic and anticancer characteristics. 

### 2.5. Polyunsaturated Fatty Acids 

Fatty acids are a type of organic molecule characterized by a lengthy hydrocarbon chain that is linked to a carboxyl group. Lipids, including fats, oils, and waxes, sourced from various organisms such as plants, animals, and microbes, exhibit a diverse range of carbon chain lengths from 1 to 30 [218,219,220]. Polyunsaturated fatty acids (PUFAs), which contain two or more double bonds in their carbon chain, are synthesized by specific desaturase enzymes. The health benefits of PUFAs have been extensively studied, including their potential to reduce the risk of chronic diseases such as heart disease, cancer, anti-inflammatory, hypolipidemic, vasodilatory, antithrombotic, and antiarrhythmic activities [196,221,222,223,224]. Vegetable oils abundant in PUFAs have garnered biomedical importance as potential dietary components for promoting normal human growth and development. LC-x-3-PUFAs are essential in regulating cholesterol synthesis, transportation, and eicosanoid synthesis, which is vital for maintaining cellular membrane integrity and overall human health. Namiki [225] proposed that combining sesamin and conjugated linoleic acid may have the potential as a weight-reducing agent. In sesame, linear acid formation and oleic desaturation ratio are limited [226].

Sesame seeds are a food source with high energy and oil content, constituting roughly 50% of the seed. Sesame oil shows a highly favorable fatty acid profile, comprising approximately 80–85% unsaturated and merely 15–20% saturated fatty acids. Sesame oil primarily contains linoleic (35–50%) and oleic (35–50%) acids, alongside minor quantities of palmitic (7–12%) and stearic (3.5–6%) acids and negligible levels of linolenic acid [227,228]. Recent scientific studies have shown that an excessive intake of n-6 fatty acids might cause a physiological condition that heightens platelet aggregation and blood clotting. Higher blood viscosity, vasospasm, vasoconstriction, and slower bleeding are all symptoms of this illness [229,230,231]. In contrast, it has been discovered that n-3 fatty acids offer several advantageous qualities, such as anti-inflammatory, antithrombotic, hypolipidemic, and vasodilatory actions. According to these results, a balanced diet with n-3 and n-6 fatty acids is essential for achieving and sustaining good cardiovascular health [232,233,234,235]. The concurrent usage of sesame and soybean oil can potentially enhance the vitamin E activity and nutritional efficacy of the lipid. Sesame oil predominantly comprises oleic and linoleic acids, constituting over 80% of the total fatty acid content [236,237]. A study was conducted by Uzun et al. [238] to investigate the variability in oil content, oil yield, and fatty acid composition among 77 sesame accessions that were gathered from various regions of Turkey. The findings indicate a significant disparity in the lipid composition of sesame accessions, with values ranging from 44.1% to 58.2%. The accession from Şanlıurfa exhibited the highest oil content, whereas the accession from Konya demonstrated the lowest. The fatty acid composition of sesame oil showed significant variability, with oleic acid being the predominant fatty acid, ranging from 32.3% to 48.2%. The results indicate that linoleic acid constituted the second most prevalent fatty acid, with a range of 36.6% to 47.4%, while palmitic acid was the most abundant, with a range of 8.3% to 9.9%. The investigation also found fluctuating quantities of additional marginal fatty acids, including stearic, arachidic, and behenic acid. The study found that the oil yield of various sesame accessions exhibited a range of 0.96 to 2.55 g per 100 seeds. Notably, the accession sourced from the Karaman region demonstrated the highest yield, while the accession obtained from the Kayseri region displayed the lowest yield. The research has identified certain favorable accessions, including a well-balanced fatty acid composition and high oil content. These accessions could be effectively employed in breeding programs to develop novel sesame cultivars with superior oil yield and quality. Therefore, based on this study, differences in the fatty acid profile of sesame seeds have been noted across various sesame cultivars. The acquired knowledge has the potential to facilitate the production of superior-grade edible oil in the forthcoming times. The germplasms of sesame originating from India exhibit a reduced concentration of saturated fatty acids, with palmitic acid being the most predominant [226]. C18:1 and C18:2 characterize Indian varieties as the primary unsaturated fatty acids. Sesame oil exhibits a considerable proportion of C18:1 and C18:2, comprising approximately 38% to 49% and 17% to 43%, respectively. Conversely, the content of C18:3 is comparatively low, measuring less than 1% [239]. A thorough evaluation of various cultivars is imperative to enhance the nutritional value of oil via manipulating the fatty acid biosynthesis pathway.

### 2.6. Short-Chain Peptides, Protein Hydrolysates, and Their Functional Properties 

Bioactive polypeptides refer to chains of amino acids connected by peptide or amide bonds and have a molecular weight that does not exceed 20 kDa. Protein fragments exhibit distinct functional roles in diverse biological, physiological, or cellular activities. Unlike naturally occurring peptides, a wide range of peptides with varying composition and function are commonly utilized using synthetically derived protein hydrolysates. According to the literature, proteins can undergo digestion by proteases, or specific fragments can be generated through bio-fermenters utilizing microbes [240,241]. According to research, sesame food preparations are a noteworthy protein source. Protein hydrolysates have extensive applications as nutritional supplements, functional ingredients, food flavor enhancers, pharmaceuticals, and cosmetics. The substances in question exhibit characteristics similar to hormones or drugs and can be categorized according to their method of operation, including but not limited to antihypertensive, antioxidative, immunomodulatory, antithrombotic, opioid, mineral binding, and antimicrobial properties [242,243,244]. Sesame seeds are rich in oil, with a content ranging from 48–55%, but they are also a significant source of protein, with the seed coat accounting for approximately 20–25% of the total dry mass [245]. Dench et al. [246] reported that defatted sesame meal, containing 40–50% protein, can serve as an appropriate source of short-chain peptides that humans can easily digest. This is attributed to the presence of sulfur-containing amino acids [247]. Defatted sesame meal has been utilized in various food products, such as biscuits containing mixed grains and fortified table bread. Bandyopadhyay and Ghosh [248] investigated using papain to produce sesame protein hydrolysates with improved functional properties, longer shelf life, and better emulsifying properties than the original sesame protein lysate. A study was conducted by Aondona et al. [249] to investigate the antioxidant and antihypertensive properties of enzymatic protein hydrolysates and ultrafiltration peptide fractions obtained from sesame seeds. The research aimed to assess the prospective application of protein hydrolysates and peptides in the mitigation and control of oxidative stress and hypertension, both of which have been associated with cardiovascular ailments. They employed enzymatic hydrolysis and ultrafiltration methodologies to produce protein hydrolysates and peptide fractions, which were subsequently subjected to in vitro assays to evaluate their antioxidant and antihypertensive characteristics. The conducted assays encompassed the determination of total phenolic content, DPPH, and ABTS radical scavenging activities and ACE and renin inhibitory activities. The study’s findings have indicated that the hydrolysates and peptide fractions of sesame seed protein exhibited noteworthy antioxidant and antihypertensive properties [249]. The hydrolysates and peptides had a total phenolic content that varied between 10.56 and 31.12 mg GAE/g protein. Additionally, their DPPH and ABTS radical scavenging activities ranged from 17.31% to 63.82% and 20.26% to 68.96%, respectively. The hydrolysates and peptides exhibited ACE and renin inhibitory activities within the range of 23.56% to 69.83% and 28.64% to 76.44%, respectively. The investigation additionally recognized the peptide fractions exhibiting the most noteworthy levels of antioxidant and antihypertensive properties. These fractions could be used as potential functional ingredients in developing nutraceuticals and functional foods [249]. They proposed that the biological functionalities of sesame seed protein hydrolysates and peptide fractions are potentially attributed to bioactive peptides, including angiotensin-converting enzyme inhibitors and antioxidants, that exhibit advantageous impacts on cardiovascular well-being. Generally, the study’s results have offered significant insights into the potential application of sesame seed protein hydrolysates and peptide fractions as functional ingredients that possess antioxidant and antihypertensive properties [249].

## 3. Processing Technology of Sesame

Different processing techniques have been found to have varying effects on the bioactive compounds of sesame seeds. Roasting the seeds has been shown to increase the oil yield and improve the antioxidant properties of the oil extract [12]. However, it has also been observed that roasting and dehulling seeds can reduce the lignan and phenolic compounds content, which are important for the antioxidant activity of sesame extracts [250]. On the other hand, processing treatments such as soaking, cooking, germination, fermentation, and microwave heating have been found to reduce the phenolic compounds and tannins content in oilseeds, including sesame seeds [251]. Overall, the processing methods used for sesame seeds can have both positive and negative effects on the bioactive compounds, and further research is needed to optimize processing techniques to maximize the retention of these beneficial compounds [252]. The conventional technology for processing sesame primarily involves mechanical pressing, aqueous extraction, and solvent methods. On the other hand, the new processing and extraction techniques for sesame encompass supercritical (subcritical) CO_2_ extraction, microwave-assisted extraction, and water enzyme extraction [253,254,255]. The biochemical characteristics of proteins can be altered through various food processing techniques, including high pressure, thermal, radiation treatments, and ultrasound. This can lead to structural changes, such as aggregation, denaturation, loss of secondary and tertiary structures, formation/disruption of different types of bonds, and chemical reactions like glycation (Maillard reactions) [256,257,258,259]. 

### 3.1. Heating Method

Various heating techniques, such as blanching, boiling, autoclaving, roasting, and frying, are frequently employed in processing sesame-based food products. Heat application elicits various structural modifications in proteins, including but not limited to cleavage and reconfiguration of protein aggregation, disulfide bonds, and chemical reactions with other constituents such as carbohydrates and lipids. The modifications above lead to alterations in the epitopes of sesame, which can decrease or increase its allergenicity [260,261,262,263]. According to research findings, boiling sesame seeds at a temperature of 100 °C for a duration of 5 min resulted in an elevation of their antigenicity. However, subsequent boiling did not exhibit any further increase in antigenicity. The study found that subjecting sesame seeds to dry roasting at 150 °C for 7.5 min increased antigenicity. Furthermore, a greater increase in antigenicity was observed when the roasting time was extended to 15 min. The application of microwave heating at 1000 W for 3 min resulted in a significant reduction in the antigenicity of sesame seeds. However, no significant alterations in antigenicity were observed when microwave heating was administered for 1 min. Studies have shown that alterations in the antigenicity of sesame seeds are associated with the method of heating employed, as well as the duration and temperature of the process [264]. This is consistent with findings from research on the thermal treatment of soybeans [261]. The protein profile of various sesame proteins undergoes distinct alterations after heat treatment [264,265]. Further investigation is required to understand the mechanism underlying alterations in antigenicity. The heat treatment process can potentially lead to the creation of advanced glycation end products, thereby augmenting the IgE binding capacity. It is imperative to consider other constituents’ influence on sesame’s allergenicity [266].

### 3.2. Mechanical Pressing

The mechanical pressing technique is a commonly employed method for extracting oil from seeds with a high oil concentration, producing exceptional quality oil. This method is characterized by simplicity, safety, and cost-effectiveness [267]. Martínez et al. [268] employed Box–Behnken designs to optimize the screw-pressing process for sesame oil extraction, resulting in a maximum oil recovery of 71.1%. The optimal conditions were achieved with a seed moisture content of 12.3%, a 4 mm restriction die, and a pressing speed of 20 r/min. The study conducted by Yin et al. [269] revealed that the volatile compounds of mechanically pressed sesame oil exhibited elevated levels of sulfur heterocyclic compounds compared to those extracted through aqueous means.

### 3.3. Aqueous Extraction

The method of aqueous extraction can extract both protein and oil simultaneously. According to Xu et al. [270] and Lv and Wu [271], this approach presents numerous benefits, such as producing superior quality oil, utilizing uncomplicated equipment and production procedures, low initial investment, and adjusting production scale. The optimal conditions for sesame oil extraction have been determined by Hou et al. [272] to include a solid-to-water ratio of 0.8 g/mL (V/m), a temperature of 70 °C, and a pH of 5.0. The circumstances mentioned earlier have led to an extraction efficiency of 82.49%. The optimization of the method by Fasuan et al. [273] resulted in improved outcomes, with oil and protein recoveries of 73.60% and 75.12%, respectively. This was achieved using a solid-to-solvent ratio of 1:3 (m/V), a pH of 11, an extraction temperature of 47 °C, and a surfactant concentration of 0.1 mol/L NaCl.

### 3.4. Aqueous Enzymatic Extraction

High oil yield and good-quality oil can be obtained through enzymatic extraction, as indicated by Liu et al. [274]. Optimal conditions for the process included a liquid-to-material ratio of 7:1 (mL/g), a microwave power of 400 W, a treatment time of 4 min, the addition of alkaline protease at 0.1% (black sesame powder), a pH of 8.0, enzymatic hydrolysis temperature of 50 °C, and a hydrolysis time of 2 h. Furthermore, de Aquino et al. [275] demonstrated that adding a certain amount of water and a suitable temperature and enzyme dosage can also promote oil production.

### 3.5. Microwave/Ultrasonic-Assisted Extraction

Microwave-assisted extraction is a technique that uses electromagnetic waves to extract substances from cells. As the temperature increases, the solvent molecules inside the cell evaporate quickly, causing pressure to build up and eventually breaking the cell wall. This leads to the rapid outflow of cell contents. Lertbuaban and Muangrat [276] used microwave-assisted extraction to extract sesamin from black sesame seeds. They identified the optimal conditions as 90% ethanol as the extractant, a solid–liquid ratio of 1:8 (g/L), a microwave power of 700 W for 9 min, and a sesamin yield of 55.48 mg. On the other hand, Sarma et al. [277] focused on the effects of solvent-based microwave-assisted extraction of sesame phenolic compounds. They found that the highest total phenolic content was 206.14 mg GAE/100 g. These studies demonstrate that the effectiveness of microwave-assisted extraction varies depending on the target substance and extraction conditions.

### 3.6. Irradiation

The antigenicity of sesame seeds and protein solutions has been the subject of numerous research studies investigating the effects of γ-irradiation and high hydrostatic pressure. The survey conducted by Zoumpoulakis et al. [278] revealed that there were no statistically significant alterations in the antigenicity of sesame proteins when white sesame seeds were subjected to irradiation at 2.5, 5.0, and 10.0 kGy, with a *p*-value of less than 0.05. The acceptable level of irradiation for food items typically falls below 10 kGy [279]. Hence, the irradiation process aimed at mitigating the allergenic properties of sesame ought to concentrate on its protein solution instead of its seeds. Increasing doses of γ-irradiation lead to a decrease in antigenicity in solutions containing Ara h 2 and Ara h 6 [280].

### 3.7. High Hydrostatic Pressure

High hydrostatic pressure is a novel technological approach that impacts non-covalent bonds, such as hydrophobic, hydrogen, ionic bonds, and salt bridges, instead of covalent bonds. This results in the denaturation of proteins and consequential structural modifications, ultimately leading to the masking or destruction of epitopes and a decrease in allergenicity. Achouri and Boye [264] observed a reduction in the antigenicity of sesame protein solutions after exposure to high hydrostatic pressure (ranging from 100 to 500 MPa) across all pH levels tested over 10 min. The decrease in antigenicity observed can be attributed to the impact of high hydrostatic pressure on the protein’s conformation, which resulted in a compact and densely packed structure that obscured the allergen epitopes. The potential decrease in antigenicity of sesame protein solution may be attributed to the diminished allergenicity of certain sesame allergens, excluding Sei i 1 and Sei 2, which could be resistant to high hydrostatic pressure owing to their disulfide bonds. 

### 3.8. Supercritical (Subcritical) Extraction

Supercritical carbon dioxide extraction is a technique that demonstrates efficacy in preserving oil’s nutritional and physiological properties while circumventing the deleterious effects of high-temperature oxidation [281,282]. Shi et al. [283] conducted a comparative analysis of sesame oil’s chemical properties, antioxidant capacity, and oxidative stability obtained through supercritical and subcritical techniques. The results of the study indicated that the processing methodologies had a negligible impact on the oil’s fatty acid and triacylglycerol composition. The suitability of compressed propane as a solvent for sesame oil extraction was demonstrated by Corso et al. [91], who found that this method required less time and pressure than carbon dioxide extraction. Liu et al. [284] optimized the extraction conditions, resulting in a 95.56% yield of sesame oil while maintaining the nutrient content.

## 4. Conclusions and Future Perspective

The comprehensive findings from recent studies highlight the numerous health advantages associated with the intake of sesame seeds, which are notably abundant in bioactive components. Lignans derived from sesame seeds offer various potential therapeutic applications, ranging from cognitive health to cardiovascular disease, cancer, and inflammation-related disorders. Functional health foods can also benefit from including tocopherols derived from sesame seeds because of their great antioxidant properties. These compounds function as antioxidants and can counteract the effects of reactive oxygen species, playing a role in safeguarding cell membranes and preventing conditions such as cancer and cardiovascular diseases. Phytosterols are also commonly incorporated into functional foods aimed at managing cholesterol levels. Numerous studies have demonstrated their ability to reduce blood cholesterol, enhance the immune system, and lower the risk of specific cancers. Although phytates are known to hinder mineral absorption, they also have potential hypocholesterolemic and anticancer properties. Numerous studies also indicate the richness of unsaturated fatty acids, mainly linoleic and oleic acid, in sesame oil, increasing its health-promoting potential. Sesame seeds, rich in oil and protein, are a valuable source of protein hydrolysates with diverse applications, including their potential as functional ingredients in nutraceuticals and functional foods, thanks to their antioxidant and antihypertensive properties as bioactive peptides. These findings underscore the significance of incorporating sesame seeds into diet and the potential for their utilization in developing dietary supplements and functional food products. Encouraging the adoption of sesame usage among consumers and food manufacturers is crucial. Additional research is essential to delve deeper into the beneficial health effects of the phytochemicals present in sesame, to understand how they work thoroughly, and to assess their clinical efficacy in treating various health conditions.

## Data Availability

The data generated from the study are clearly presented and discussed in the manuscript.

## Abbreviations

ABCA1	Binding cassette transporter A1
ABTS	2,2’-azino-bis(3-ethylbenzothiazoline-6-sulfonic acid), radical cation
ACC	Acetyl-CoA carboxylase
ACE	Antioxidant capacity equivalent
AD	Alzheimer’s disease
ALT	Alanine transaminase
AMPK	AMP-activated protein kinase
ARE	Antioxidant response element
AST	Aspartate transaminase
Aβ	Amyloid-β
BBB	Blood–brain barrier
BCAAs	Branched-chain amino acids
Bcl-2	B-cell lymphoma 2
BDNF	Brain-derived neurotrophic factor
BUN	Blood urea nitrogen
CAT	Catalase
C57BL/6J	Strain of mice used as a universal model for studying diet-induced obesity
CD36	Scavenger receptor involved in cholesterol uptake
CDKs	Cyclin-dependent kinases
CKD	In chronic kidney disease
COX-2	Cyclooxygenase-2
DHT	Dihydrotestosterone
DNA	Deoxyribonucleic acid
DPPH	2,2-diphenyl-1-picrylhydrazyl, radical
ESRD	End-stage renal disease
ERK1/2	Extracellular signal-regulated kinase 1/2
ER	Endoplasmic reticulum
FAO	Food and Agriculture Organization
FAS	Fatty acid synthase
FcεRI	The high-affinity receptor for the Fc region of immunoglobulin E
FoxO	Forkhead box O
GAE	Gallic acid equivalent
GSH-Px	Glutathione peroxidase
HDL-C	High-density lipoprotein cholesterol
HepG2	Human liver cell line
HMG-CoA reductase	3-hydroxy-3-methylglutaryl-CoA reductase
HO-1	Heme oxygenase-1
IgE	Immunoglobulin E
IKKα	Kinase α
IL-1	Interleukin 1
IL-1β	Interleukin-1 beta
IL-10	Interleukin-10
IL-6	Interleukin 6
iNOS	Nitric oxide synthase
JNK	c-Jun N-terminal kinase
LCAT	Lecithin:cholesterol acyltransferase
LDL	Low-density lipoprotein
LDL-C	Low-density lipoprotein cholesterol
LOX	Lipoxygenase
LPS	Lipopolysaccharides
LXRα	Liver X receptor alpha
MAPK	p38 mitogen-activated protein kinase
MDA	Malondialdehyde
MMP	Mitochondrial membrane potential
mTOR	Mechanistic target of rapamycin
MTT	3-(4,5-dimethylthiazol-2-yl)-2,5 diphenyl tetrazolium bromide
NAFLD	Non-alcoholic fatty liver disease
NF-κB	Nuclear factor kappa-light-chain-enhancer of activated B cells
Nrf2	Nuclear factor erythroid 2-related factor 2
NSCLC	Non-small cell lung cancer
p53	Tumor protein, regulatory protein that is often mutated in human cancers
PI3K-PKB	Signaling pathway
PPAR-γ	Peroxisome proliferator-activated receptor gamma
PUFAs	Polyunsaturated fatty acids
PXR	Pregnane X receptor
ROS	Reactive oxygen species
SAMP8	Senescence-accelerated mouse-prone 8
SCFAs	Short-chain fatty acids
SOD	Superoxide dismutase
SR-A	Scavenger receptor class A
SR-BI	Scavenger receptor class B type I
SREBP-1c	Sterol regulatory element-binding protein 1c
SIRT1	Sirtuin 1
TLR4	S gene-encoding Toll-like receptor 4 in humans
TNF-α	Tumor necrosis factor-alpha
UVB	Ultraviolet B
